# Factors affecting face mask-wearing behaviors to prevent COVID-19 among Thai people: A binary logistic regression model

**DOI:** 10.3389/fpsyg.2022.996189

**Published:** 2022-11-08

**Authors:** Wonpen Kaewpan, Kunwadee Rojpaisarnkit, Supa Pengpid, Karl Peltzer

**Affiliations:** ^1^Department of Public Health Nursing, Faculty of Public Health, Mahidol University, Bangkok, Thailand; ^2^Department of Public Health, Faculty of Sciences and Technology, Rajabhat Rajanagarindra University, Chachoengsao, Thailand; ^3^Department of Health Education and Behavioral Sciences, Faculty of Public Health, Mahidol University, Bangkok, Thailand; ^4^Department of Public Health, Sefako Makgatho Health Sciences University, Pretoria, South Africa; ^5^Department of Psychology, University of the Free State, Bloemfontein, South Africa; ^6^Department of Psychology, College of Medical and Health Science, Asia University, Taichung, Taiwan

**Keywords:** face mask, mask-wearing behaviors, COVID-19, disease prevention behavior, health behavior, binary logistic regression

## Abstract

**Objectives:**

Face mask wearing is a standard preventive measure, in addition to handwashing and physical distancing. Individuals may find that wearing a face mask protects their physical health and prevents viral transmission. However, none of the studies in Thailand identified factors associated with face mask-wearing behaviors among Thai people. Therefore, this study aims to determine factors affecting face mask-wearing behaviors to prevent COVID-19.

**Methods:**

This research is analytical survey research. The data used in this study were under the project title “The assessment of psychosocial and behavioral response and compliance to restriction measures to prevent and control COVID-19: A series of the rapid survey.” A total of 6,521 people participated in an online survey by multi-stage sampling. Bivariate logistic regression analysis was used to examine the factors associated with face mask-wearing behaviors.

**Results:**

After adjusting for independent variables (i.e., gender, age, education, career, smoking, and comorbidity disease), the bivariate logistic regression analysis revealed that gender, age, and career were statistically significant to the face mask-wearing behaviors (*p* < 0.05). Level of education, smoking, and comorbidity disease were not statistically significant with face mask-wearing behaviors among Thai people.

**Conclusion:**

Further study should explore broader on individual face mask perceptions and wearing in the continuing of COVID-19 across gender, age, and careers to better understand their health behaviors and to inform further policy. In addition, the development of an intervention to promote face mask wearing should target men who age below 30 yrs. and did not work in government services careers as this group of the population was likely not to wear a face mask outside the home.

## Introduction

Coronavirus disease (COVID-19) is an acute respiratory infectious disease (Mboowa et al., 2021). COVID-19 was identified in December 2019, with the first confirmed case of COVID-19 infection in Wuhan, China. Later in March 2020, World Health Organization declared the COVID-19 outbreak as a global pandemic (Zhang et al., 2020). An estimated 609 million people worldwide have been infected with COVID-19, with 6.50 million deaths in September 2022 (Worldometers, 2022). After the COVID-19 outbreak in China, Thailand was the first country that identifies a confirmed case of COVID-19 infection. People in Thailand were encouraged to protect themselves and their communities by applying basic health measures such as mask wearing, handwashing, and physical distancing to prevent COVID-19 transmission (World Health Organization, 2020). Thai people affirmatively responded to wearing face masks to prevent viral transmission from both symptomatic and asymptomatic individuals (Rojpaisarnkit et al., 2022). Since the outbreak of coronavirus, wearing a face mask outside the home has become mandatory, and it is a new behavior among Thai people. The general population was advised to wear face masks as a mechanical barrier to prevent the dispersion of droplets, in adjunction to physical distancing, and increase the frequency and consistency of handwashing by using hand sanitizers such as soap or alcohol-based solutions (Centers for Disease Control Prevention, 2022). Against the spread of COVID-19, wearing a face mask policy has been employed in the COVID-19 pandemic plans as a public and personal health control measure (Ganczak et al., 2021). A face mask is an effective tool in preventing the spread of COVID-19 (Haischer et al., 2020). Several studies have investigated the importance of wearing face masks. Studies found that wearing face masks was strongly associated with a significant decrease in the risk of respiratory infections. In contrast, no association was found between decreasing the risk of respiratory infections and hand hygiene or physical distancing (Rabie and Curtis, 2006; Chu et al., 2020; Doung-Ngern et al., 2020; Natnael et al., 2021). Even though the World Health Organization (2020) recommends face masks used in the community for asymptomatic individuals in the pandemic to reduce transmission in the community, some people still resist wearing them.

Previous studies have examined the factors that influence individual mask-wearing behaviors. Studies found that different individual characteristics significantly affect individual mask-wearing behaviors. For example, gender was found in four studies (Chuang and Liu, 2020; Haischer et al., 2020; Annaka, 2021; Looi et al., 2021). Age was found in four studies (Haischer et al., 2020; Knotek et al., 2020; Laksono et al., 2020; Howard, 2021). Level of education was found in two studies (Laksono et al., 2020; Sikakulya et al., 2021). Cigarette smoking was found in two studies (Klein et al., 2021; Sun et al., 2021). Career was found in one study (van den Broek-Altenburg et al., 2021). Even though none of the studies found an association between mask-wearing behaviors and comorbidity disease, comorbidity disease is the significant factor associated with the severity of coronavirus cases (Ejaz et al., 2020; Sanyaolu et al., 2020; Kompaniyets et al., 2021).

None of the studies have examined the factors influencing mask-wearing behaviors among Thai people. However, wearing a face mask is a useful and low-cost strategy adjunct to handwashing and physical distancing during the COVID-19 pandemic (Coclite et al., 2020). Therefore, understanding individual mask-wearing behaviors will be beneficial to support face mask use in a community to respond to the pandemic. Thus, this study aimed to identify factors influencing individual mask-wearing behaviors using a logistic regression model.

## Methods

This research is analytical survey research. The data used in this study were the data under the project title “The assessment of psychosocial and behavioral response and compliance to restriction measures to prevent and control COVID-19: A series of the rapid survey.” The results of a cross-sectional study have been published elsewhere. A total of 6,521 people participated in an online survey by multi-stage sampling (three stages were stratified randomly from the region, province, and residential area) from March to June 2020.

### Measures

The questionnaire was developed based on a literature review of research and theories to assess psychosocial factors and behavioral responses, and compliance with the restriction measures to prevent and control the transmission of COVID-19 (Rojpaisarnkit, 2016; Thomas et al., 2020; Fakhira et al., 2021; Latkin et al., 2021; Rojpaisarnkit et al., 2022). The questionnaire used in this study included six questions related to personal attributes and one question related to mask-wearing behaviors. These questions were part of the large study “The assessment of psychosocial and behavioral response and compliance to restriction measures prevent and control COVID-19: A series of the rapid survey.” In addition, five experts examined the content validity and reliability of the questionnaire, and Cronbach's alpha scores ranged from 0.802 to 0.966.

### Statistics

All data were downloaded from Google forms for data analysis. Descriptive statistics were reported as mean, standard deviation (SD), and percentages to overview the sample's characteristics. Testing the data for normal distribution was done by the Kolmogorov–Smirnov test statistic. Binary logistic regressions were employed to identify factors associated with face mask-wearing behaviors with selective explanatory variables. The dependent variable was wearing a face mask (vs. not wearing a face mask). Independent variables were selected based on previous literature about face mask-wearing behaviors, including gender, age, level of education, career, smoking, and comorbidity disease. In this study, independent variables were treated as categorical. Before the data analysis, the assumption of multicollinearity was tested, and there was an absence of collinearity (*r* < 0.8) (Kim, 2019).

The binary logistic regression model is given below:


(1) logit(π)=β0+β1X1+β2X2+…+βpXp

where *β*0, *β*1, …, *βp* are regression parameters, *X*1, *X*2,…, *Xp* are explanatory variables, and π is the probability of success.

The steps in the analyses using the binary logistic regression were (1) calculating descriptive statistics, (2) estimating regression parameters using maximum likelihood estimation, (3) conducting the likelihood ratio test, (4) conducting the Wald test (The Wald values are obtained by dividing the slope coefficients by their standard error. If the null hypothesis is true, the Wald value has an approximate standard normal distribution for a large sample. The null hypothesis is rejected if the Wald value is greater than the critical standard normal value or the *p*-value is less than the significance level) (Berhie and Gebresilassie, 2016), (5) determining the final model, (6) interpreting the regression coefficients and the odds ratio, and (7) evaluating the goodness of fit of the model using the accuracy of classifications.

A Chi-square test for independence was conducted to test the relationship between face masks wearing behaviors and individual explanatory variables.

The null hypothesis is that the two categorical variables are independent (or there is no relationship between the two categorical variables).

The alternate hypothesis is that the two categorical variables are dependent (or there is a relationship between the two categorical variables).

When the null hypothesis is rejected, there is evidence of an association between the two variables.

Thus, for face mask-wearing behaviors, the hypotheses are as follows:

(i) H_0_: A respondent's face mask-wearing behaviors are independent or unrelated; and
(ii) H_1_: A respondent's face mask-wearing behaviors are related (dependent on each other).

An odd ratio was applied to analyze the probability of wearing a face mask and not wearing a face mask. Model suitability analysis was done using the Hosmer and Lemeshow statistics test (Hosmer and Lemeshow, 2000). Assessment of the validity of the methods, results, and interpretations of the data were carried out in consultation with the research experts.

## Results

### Descriptive statistics

Table 1 presents sample characteristics. Among 6,521 participants included in the analysis, most respondents wear a face mask outside the home (*n* = 6,362, 97.6%). Among this population, most of them were female (*n* = 4,130, 64.9%), age between 40 and 49 years (*n* = 1,563, 24.6%), completed bachelor's degree (*n* = 3,265, 51.3%), work in government services (*n* = 2,368, 37.2%), and non-smoking (*n* = 5,637, 97.8%). Among the sample who did not wear a face mask outside the home (*n* = 90, 56.6%), most of them were male (*n* = 90, 56.6%), age between 20 and 29 years (*n* = 50, 31.4%), completed bachelor's degree (*n* = 74, 46.5%), and non-smoking (*n* = 125, 95.5%).

**Table 1:** Frequency, percentage, and chi-square for the categorical explanatory variables.

**Variables**	**Wearing a face mask outside the home** **(*N* = 6,362) (*n*, %)**	**Not wearing a face mask outside the home** **(*N* = 159) (*n*, %)**	**df**	**Chi-square** **(*p*-value)**
**Gender**			1	33.333 (< 0.001)
Male	2,232 (35.1)	90 (56.6)		
Female	4,130 (64.9)	69 (43.4)		
**Age** (yrs.)			5	75.095 (< 0.001)
**Mean** **±SD (range)**	40.22 ±12.80 (15–100)	35.80 ±16.20 (15–80)		
15–19	291 (4.6)	25 (15.7)		
20–29	1,175 (18.5)	50 (31.4)		
30–39	1,548 (24.3)	41 (25.8)		
40–49	1,563 (24.6)	26 (16.4)		
50–59	1,416 (22.3)	13 (8.2)		
Above 60	369 (5.8)	4 (2.5)		
**Education**			3	25.482 (< 0.001)
Primary school	191 (3.0)	12 (7.5)		
Secondary school	1,338 (21.0)	50 (31.4)		
Bachelor's degree	3,265 (51.3)	74 (46.5)		
Postgraduate	1,568 (24.6)	23 (14.5)		
**Career**			5	34.774 (< 0.001)
Unemployment	907 (14.3)	43 (27.0)		
Private business	742 (11.7)	17 (10.7)		
General employee/artisan	716 (11.3)	25 (15.7)		
Government services	2,368 (37.2)	35 (22.0)		
Farmers/agricultures	105 (1.7)	6 (3.8)		
Employee in organization	1,524 (24.0)	33 (20.8)		
**Cigarette smoking**			1	15.046 (< 0.001)
Yes	125 (2.2)	34 (4.5)		
No	5,637 (97.8)	125 (95.5)		
**Comorbidity disease**			1	4.476 (0.034)
Yes	1,225 (19.1)	20 (12.6)		
No	5,137 (80.9)	139 (87.4)		

### Bivariate analysis

The Chi-square test was used to examine the association between dependent and independent variables. The binary logistic regression model will include the variables that significantly affect face mask-wearing behaviors. Gender, age, level of education, career, cigarette smoking, and comorbidity disease were significantly associated with face mask-wearing behaviors at a 5 % significance (*p*-values <0.05) presented in Table 1.

### Binary logistic regression analysis

Table 2 presents binary logistic regression analysis. The Wald test was used to test the set of hypotheses (H_0_: β*r* = 0vs H_1_: β*r* ≠ 0) for individual regression slope coefficients. The Wald tests suggested that gender, age (30 yrs. and above), and career (government services) were statistically significant at 0.05 on each variable.

**Table 2:** Binary logistic regression results.

**Variables^a^**	**B**	**SE**	**Wald**	**df**	***p*-value**	**Odds ratio**	**95% CI for odds ratio**	
							**Lower**	**Upper**
**Gender** (male vs. female)	0.846	0.175	23.220	1	0.000	2.330	1.652	3.286
**Age** (yrs.) (vs. age 15–19)			31.237	5	0.000			
20–29	0.474	0.298	2.535	1	0.111	1.607	0.896	2.880
30–39	0.804	0.321	6.288	1	0.012	2.235	1.192	4.192
40–49	1.225	0.348	12.411	1	0.000	3.403	1.722	6.727
50–59	1.830	0.401	20.831	1	0.000	6.234	2.841	13.681
Above 60 yrs.	1.931	0.578	11.152	1	0.001	6.899	2.221	21.435
**Education** (vs. primary school)			0.866	3	0.834			
Secondary school	0.316	0.340	0.863	1	0.353	1.371	0.704	2.670
Bachelor	0.258	0.360	0.516	1	0.473	1.295	0.640	2.621
Postgraduate	0.272	0.417	0.426	1	0.514	1.313	0.580	2.971
**Career** (vs. unemployment)			8.101	5	0.151			
Private business	0.398	0.307	1.688	1	0.194	1.489	0.817	2.716
General employee artisan	0.337	0.273	1.524	1	0.217	1.400	0.821	2.390
Government services	0.618	0.272	5.152	1	0.023	1.855	1.088	3.163
Farmers agricultures	−0.343	0.461	0.555	1	0.456	0.709	0.287	1.751
Employee in organization	0.521	0.266	3.842	1	0.050	1.684	1.000	2.837
**Cigarette smoking** (vs. not smoking)	−0.155	0.227	0.467	1	0.494	0.856	0.548	1.337
**Comorbidity disease** (vs. no comorbidity disease)	−0.171	0.253	0.455	1	0.500	0.843	0.514	1.384
Constant	1.821	0.435	17.517	1	0.000	6.180		

^a^Variable(s) entered in step 1: Gender, age, education, career, smoking, and comorbidity disease.

In general, women were more likely to report wearing face masks than men (*p* < 0.001, OR = 2.330, 95% CI: 1.652–3.286). The probability of wearing face masks increased significantly as the age increased. The results showed that age of 60 and above has more contributions than other factors (*p* = 0.001, OR = 6.899, 95% CI: 2.221–21.435), followed by the age of 50–59 (*p* < 0.001, OR = 6.234, 95% CI: 2.841–13.681), age of 40–49 (*p* < 0.001, OR = 3.403, 95% CI: 1.722–6.727), and age of 30–39 (*p* = 0.012, OR = 2.235, 95% CI: 1.192–4.192), respectively. Respondents who worked in government services were significantly associated with a higher probability of wearing a face mask compared to other careers (*p* = 0.023, OR = 1.855, 95% CI: 1.088–3.163), followed by an employee in a private organization (*p* = 0.050, OR = 1.684, 95% CI: 1.000–2.837).

A good model is a model which has a minimal chance of misclassification (Hosmer and Lemeshow, 2000). The method used in the binary classification is a Fisher linear discriminant analysis. Table 3 presents the results of the classification accuracy using this method. The total percentage of accuracy is 97.60%, which indicates a good model.

**Table 3:** Classification accuracy.

**Observed^a^**	**Predicted**		**Total**	**% Accuracy**
	**Not wear mask (no)**	**Wear mask (yes)**		
Not wearing a mask	155	4	159	97.48
Wearing a mask	153	6,209	6,362	97.60
Total	308	6,213	6,521	97.60

^a^The cutoff value is 0.50.

## Discussion

The result revealed that 97.6% of Thai people wear a face mask outside the home, which is consistent with the study of Zhang et al. (2021) which found that 66% of Malaysian people (*n* = 708) always wear masks in public places. Conversely, the study in the USA found that 71.2% of Latino population never wear masks, and 97.3% of them stated they have a low risk of getting an infection (Karout et al., 2020). Although it is clear that wearing a mask is useful in preventing the spread of COVID-19, the study also recommended that to prevent respiratory infections effectively, wearing a face mask should be done in combination with other preventive measures like ventilation and distancing (Cheng et al., 2021).

This study described factors that influence face mask-wearing behaviors. The model indicated that gender, age, and career were significantly associated with face mask-wearing behaviors (*p* <0.05). In contrast, the level of education, smoking, and comorbidity disease were not associated with face mask-wearing behaviors.

Women were more likely to wear face masks compared to men. This finding is consistent with the result of previous studies that men are less likely to wear face masks and more likely to exhibit unacceptable face mask-wearing practices during the COVID-19 pandemic (Chuang and Liu, 2020; Gunasekaran et al., 2020; Looi et al., 2021; Hearne and Niño, 2022). In addition, men may feel that their independence is less threatened by COVID-19; thus, they are less likely to wear face masks (Howard, 2021). A study in Bangladesh found that men perform lower level of preventive COVID-19 behaviors (Hosen et al., 2021).

A study in Philippines found that women had good preventive behavior (Arceo et al., 2021). In Malaysia, women who washed their hands frequently and reported having more personal protective equipment were more likely to wear masks (Zhang et al., 2021). Moreover, most women wear face masks correctly (Ganczak et al., 2021) and do not reuse surgical masks (Pereira-Ávila et al., 2020). The study on Egyptian women found they had the highest values on anxiousness, avoiding, and cleaning (Aboul-ata and Qonsua, 2022). Further study should explore broader on individual face mask perceptions and wearing as well as the role of gender in the relations.

Older people aged 60 and above wear face masks more than other age groups, consistent with the study in Mexico's adults that older people had better preventive health behavior than other age groups (Sánchez-Arenas et al., 2021), which also supported that individuals wearing a mask increased significantly with age (Haischer et al., 2020). In addition, a study in Thailand and Philippines found that age was one of the most influenced COVID-19 prevention behaviors (Arceo et al., 2021; Rojpaisarnkit et al., 2022). Moreover, age significantly correlated with self-defense behaviors from COVID-19 (Choorust et al., 2022) and was a predictor of wearing a face mask in Indonesian people (Laksono et al., 2020).

Respondents who work in government services were significantly associated with a higher probability of wearing face masks than in other careers, consistent with Laksono et al. (2020), that found work types were predictors of wearing a face mask behavior in Indonesian people. One of the possible reasons why government services workers are more likely to wear face masks outside the home is that they are more obedient to government policy than other work types. Moreover, the study in the USA found that employment status was associated with COVID-19 preventive behaviors (Li et al., 2020). Professional workers in Mexico had better preventive health behavior compared with other work types (Sánchez-Arenas et al., 2021).

Level of education, cigarette smoking, and comorbidity disease were not significantly associated with face mask wearing. Education was not associated with face mask wearing which is consistent with the study by Loleka and Ogawa (2022) in Congo that found no significant difference in the level of education on women's practices to control the transmission of COVID-19. However, a study in Bangladesh found that people with no formal education had lower performing preventive COVID-19 behaviors (Hosen et al., 2021). In accordance with an Indonesian study, people who had higher educational levels were more inclined to engage in good preventive behavior (Husnah et al., 2021).

Cigarette smoking was not associated with face mask wearing, whereas a study in Bangladesh found that smoking cigarettes reported lower performing preventive COVID-19 behaviors (Hosen et al., 2021). Nevertheless, Klein et al. (2021) study found that tobacco users significantly changed their tobacco use routines during the COVID-19 pandemic. In addition, few smokers mentioned quitting cigarette smoking due to the pandemic. Therefore, further studies should explore the relationship between face mask-wearing behaviors, changes in tobacco use, and the decision to quit smoking to understand the factors associated between smoking and wearing face mask behaviors.

Comorbidity diseases were not associated with face mask wearing, in contrast to the finding in an Indonesian study that urban residents with the underlying diseases had poor preventive behavior (Husnah et al., 2021). In addition, people with underlying diseases in Iran had higher levels of fear of COVID-19 (Mosazadeh et al., 2021), and it is due to the COVID-19 behavioral awareness.

In addition, the binary logistic regression model suggested that gender, age, and career were significantly associated with face mask-wearing behaviors. The probability of wearing a face mask increased in women aged over 30 years and working in government services. Interestingly, older people frequently wear face masks more than other age groups. The results of this study will be helpful in policy planning to improve strategies to prevent COVID-19 transmission among individuals with different personal characteristics, especially in gender, age, and career.

## Limitations and strengths of the study

Face mask-wearing behavior may depend on the current policies of COVID-19 prevention in each country. Policies may change based on the epidemic situations which may be resulting in different factors that affect face mask-wearing behavior. For example, this study was conducted during the second wave of the COVID-19 outbreak in Thailand, and during that time, Thailand had strict COVID-19 prevention measures, including the mandatory use of face masks. Recently, the Ministry of Public Health of Thailand has announced that wearing masks is now voluntary but advised people to wear masks if in crowded settings or had an underlying disease (The Secretariat of the Cabinet, Thailand, 2022), while the strengths of the study are a many number of participants and data collection conducted during the second wave of the COVID-19 outbreak in Thailand, which represent disease prevention behaviors among Thai people during the outbreak of communicable diseases. Factors affecting disease preventive behaviors can be used as important information in designing people's behavioral promotion in epidemic prevention.

## Data availability statement

The original contributions presented in the study are included in the article/supplementary materials, further inquiries can be directed to the corresponding author.

## Ethics statement

The studies involving human participants were reviewed and approved by Faculty of Social Sciences and Humanities, Mahidol University [Cert. no. MUSSIRB 2020/127(B1)]. The patients/participants provided their written informed consent to participate in this study.

## Author contributions

WK, KR, SP, and KP conceived and planned the study, and carried out the data collection. KR contributed to the interpretation of the results. WK and KR took the lead in writing the manuscript. All authors provided critical feedback and helped shape the research, analysis and manuscript.

## Funding

Scholarly publishing was supported by the Faculty of Public Health, Mahidol University.

## Conflict of interest

The authors declare that the research was conducted in the absence of any commercial or financial relationships that could be construed as a potential conflict of interest.

## Publisher's note

All claims expressed in this article are solely those of the authors and do not necessarily represent those of their affiliated organizations, or those of the publisher, the editors and the reviewers. Any product that may be evaluated in this article, or claim that may be made by its manufacturer, is not guaranteed or endorsed by the publisher.
